# Identification and distribution of *Rhipicephalus microplus* in selected high-cattle density districts in Uganda: signaling future demand for novel tick control approaches

**DOI:** 10.1186/s12917-024-03979-z

**Published:** 2024-03-25

**Authors:** Patrick Etiang, Stella A. Atim, Joseph Nkamwesiga, David Nalumenya, Charles Byaruhanga, Steven Odongo, Patrick Vudriko, Anna Rose Ademun, Savino Biryomumaisho, Joseph Erume, Charles Masembe, Emma C. Thomson, Dennis Muhanguzi, Robert Tweyongyere

**Affiliations:** 1 College of Veterinary Medicine, Animal Resources and Biosecurity (COVAB), Makerere University, P.O. Box 7062, Kampala, Uganda; 2 Faculty of Agriculture and Animal Sciences, Busitema University, P.O. Box 236, Tororo, Uganda; 3 Ministry of Agriculture, Animal Industry and Fisheries, P.O. Box 102, Entebbe, Uganda; 4 National Agricultural Research Organization, P.O. Box 259, Entebbe, Uganda; 5 College of Natural Sciences (CONAS), Makerere University, P.O. Box 7062, Kampala, Uganda; 6 MRC-University of Glasgow Centre for Virus Research (CVR), Glasgow, United Kingdom; 7 Vectors and Vector-Borne Diseases Research Programme, Department of Veterinary Tropical Diseases, Faculty of Veterinary Science, University of Pretoria, Private Bag X04, Onderstepoort, 0110 South Africa

**Keywords:** Uganda, Ticks, Cattle, Morpho-taxonomic keys, *12S* rRNA, *16S* rRNA, ITS2, *R. microplus*, Tick-borne diseases

## Abstract

**Background:**

*Rhipicephalus* (*Boophilus*) *microplus* (Canestrini, 1888), the Asian blue tick, is a highly invasive and adaptable ectoparasite. This tick species has successfully established itself in most regions of the world, with movement of cattle being a major driver for its spread. In the recent past, *R. microplus* ticks have been reported in three districts of Uganda. Information on its spread and distribution are vital in deepening our understanding of the ecological scenarios that lead to tick persistence and in the formulation of control strategies. This is especially important in the cattle-dense districts.

**Methods:**

We randomly collected tick specimens from 1,461cattle spread across seven cattle dense districts located in the Central, Karamoja and West Nile regions of Uganda from January to September 2020. The ticks were identified using standard morpho-taxonomic keys and the *R. microplus* tick species identities were confirmed by sequencing of the ITS2 region, *12S* rRNA and *16S* rRNA genes and phylogenetic analyses.

**Results:**

Adult ticks (*n* = 13,019) were collected from 1,461 cattle. Seventeen tick species were identified based on morpho-taxonomic keys and the majority (47.4%; *n*=6184) of these were *R. appendiculatus*. In total, 257 *R. microplus* ticks were found infesting cattle in 18 study sites in the districts of Amudat, Kaabong, Napak (Karamoja region) and Arua (West Nile region). The identity of *R. microplus* was confirmed using molecular technics. No *R. microplus* tick was recorded in the districts of Lyantonde and Nakaseke (Central region). Arua district accounted for 82.1% (*n*=211) of the *R. microplus* ticks recorded followed by Napak district at 16.3% (*n*=42), while Amudat and Kaabong districts accounted for 1.5% (*n*=4).

*Rhipicephalus microplus* and *R. decoloratus* co-existed in 6 of the 13 study sites in Arua district, while in another 6 study sites, no *R. decoloratus* was recorded. In the Karamoja region districts *R. decoloratus* co-existed with *R.microplus*. Of the total 618 ticks belonging to four species of the subgenus *Boophilus* recorded in this study, *R. decoloratus* accounted for 50.04% (*n*=334), followed by *R. microplus* at 41.58% (*n*=257), *R. geigyi* at 2.75% (*n*=17) and *R. annulatus* at 1.61% (*n*=10). In the districts of Amudat, Kaabong and Napak, *R. decoloratus* was more dominant (76.1%; *n*=179) of the three *Rhipicephalus* (*Boophilus*) tick species recorded, followed by *R. microplus* (19.5%; *n*=46) and *R. geigyi* (4.2%; *n*=10). Contrariwise, *R. microplus* was more dominant (84%; *n*=211) in Arua district followed by *R. decoloratus* (10.7%; *n*=27), *R. annulatus* (3.9%; *n*=10) and *R. geigyi* (1.1%; *n*=3). Phylogenetic analyses of the ITS2 region, *12S* rRNA and *16S* rRNA genes revealed subgrouping of the obtained sequences with the previously published *R. microplus* sequences from other parts of the world.

**Conclusion:**

*Rhipicephalus microplus* ticks were found infesting cattle in four districts of Uganda. The inability to find *R. decoloratus*, an indigenous tick, from six sites in the district of Arua is suggestive of its replacement by *R. microplus*. *Rhipicephalus microplus* negatively affects livestock production, and therefore, there is a need to determine its distribution and to deepen the understanding of the ecological factors that lead to its spread and persistence in an area.

**Supplementary Information:**

The online version contains supplementary material available at 10.1186/s12917-024-03979-z.

## Background

The African blue tick, *Rhipicephalus* (*Boophilus*) *decoloratus* and the Asian blue tick, *Rhipicephalus* (*Boophilus*) *microplus* are the two economically important *Rhipicephalus* (*Boophilus*) tick species infesting cattle in sub-Saharan Africa [1]. *Rhipicephalus decoloratus* is indigenous to the African continent, while *R. microplus* was most probably introduced through cattle importation [2]. Uganda has a wide diversity of tick species infesting domestic animals [3–7]; however, traditionally *R. microplus* has not featured greatly [8, 9]. Cattle movement is thought to be the major driver of the spread of *R. microplus* both locally and globally [2, 10, 11].

*Rhipicephalus* (*Boophilus*) *microplus* (Canestrini, 1888), is a highly invasive and adaptable ectoparasite that has established itself in most of the world’s regions where cattle are kept [10–13]. The efficiency of the rate of spread of *R. microplus* from its area of discovery is well-known [2]. In West Africa, within a period of 10 years since its detection [14], the tick had spread to another 7 countries [15–17], and successfully replaced the indigenous *R. decoloratus* [2]. In East Africa, the tick was introduced from Asia through the importation of cattle [2] and it has been reported in the coastal areas of Kenya and Tanzania [18, 19], north Tanzania [20] and Equatorial province of South Sudan [21, 22]. The reports in north Tanzania and South Sudan are a major risk of spread of *R. microplus* to Uganda. Much as its distribution is not well understood due to a limited number of country-wide tick surveys, it could be replacing *R. decoloratus* in some of the areas where it has been reported. In Uganda, *R. microplus* had never been recorded prior to a study [23] conducted in the southeastern Serere district, where it had successfully replaced the indigenous *R. decoloratus*. Presence of the tick has also previously been confirmed amongst cattle from Soroti and Gulu districts in the eastern and northern regions of Uganda respectively [6]. In spite of the reports about *R. microplus* in Uganda, no evidence of *Babesia bovis*, one of the transmitted pathogens, had been recorded in Uganda until 2023 [24].

The introduction and spread of *R. microplus* and *B. bovis* to Uganda from potential risk areas like South Sudan, Kenya and Tanzania can cause a negative impact on the cattle industry. *Rhipicephalus microplus* is an efficient vector of *B. bovis*, the more pathogenic cause of babesiosis. The tick has a tendency to replace other tick species of the same sub-genus [2, 11, 25–27] and is associated with rapid development of resistance to most of the acaricides [2, 28]. Uganda has registered acaricide resistance in a number of tick species [29], mainly caused by over-use and misuse of acaricides [30, 31]. Therefore, there is potential for development of acaricide resistance in *R.microplus* as well [32].

This study was undertaken to determine the possibility of the silent existence of *R. microplus* in other cattle keeping districts of Uganda apart from the districts of Serere, Gulu and Soroti. Understanding of the local tick species diversity, their distribution, life cycles and the level of susceptibility of their hosts can support the implementation of effective measures to control ticks and the pathogens they vector [33]. Therefore, the main objectives of the present study were to confirm the presence of *R. microplus* in the seven high cattle density districts of Uganda, determine the geographic distribution of *R. microplus* in seven districts with reference to the previously known ticks in this country, and make an attempt to determine the relative number of *R. microplus* in relation to *R. decoloratus* so as to establish whether *R. decoloratus* has been displaced.

## Methods

### Study design and sites

This study was undertaken in seven districts of Uganda. Uganda is stratified into five administrative levels (districts, counties/municipalities, sub counties/town councils, parishes/wards, and villages/cells) and a collection of districts make a region. Three regions of Karamoja, West Nile and Central were purposively selected for this study because of their known high cattle density and therefore they could be areas of high tick infestation. Cattle included in this survey were randomly selected from herds, irrespective of gender, breed, or age, and provided they had not undergone acaricidal treatment in the past 30 days.

In Karamoja region, four districts of Amudat, Kaabong, Kotido, and Napak were randomly selected. Each district had eight sampling sites totaling to 32 sites. Livestock rearing, with cattle as the leading type, is the key livelihood activity in Karamoja region [34, 35]. Karamoja region has 2.3 million (19.8%) of the national cattle population of 11,408,750 [35], and Kotido district registered the highest cattle herd estimated at 6.1% (694,250) of the national herd of 11,408,750 [35]. Besides their economic importance, cattle are significant in the social and cultural life of the Karimojong pastoralists [34, 36]. Karamoja region is in northeastern Uganda and it is bordered by South Sudan to the north, Kenya to the east, and the sub-regions of Acholi and Teso to the west, and Bugisu to the south. The area is semi-arid with an average annual rainfall ranging from 300mm in the pastoral regions to 1200 mm in western areas of Abim and Nakapiripirit. The average annual temperature ranges from 16 ^0^C in the highlands to 24 ^0^C in the lowlands. Most cattle were classified under communal grazing with a slight element of transhumance.

In West Nile sub-region, only Arua district with 13 sampling sites was selected. This sub-region of northern Uganda is comprised of about 7.2% (829,204) of the national cattle herd of 11,408,750. Arua district has an estimated 1% (117,157) of the national cattle herd [35]. West Nile sub-region is located in north-western Uganda. It is bordered by South Sudan to the north, Democratic Republic of Congo to the west and south, and by the Albert Nile to the east. Arua district has a tropical savannah climate and an average annual rainfall ranging from 300 to 1200 mm. The average annual temperature ranges from 19 °C in rainy season to 30 °C in dry season, while, the annual rainfall average ranges from 592 to 1210 mm. Majority of the cattle are classified under communal grazing.

In Central region, two districts of Lyantonde (8 sampling sites) and Nakaseke (13 sampling sites) were selected. These districts are located in the central cattle corridor region, stretching from the south west to the north eastern part of Uganda. Nakaseke district has about 1.4% (160,737) of the national cattle herd of 11,408,750 while Lyantonde district has about 0.6% (68,572) of the national cattle herd [35]. Central region is located in central Uganda bordering western region to the west and south, Busoga sub-region and Lake Victoria to the East and Lake Kyoga to the north. The climate is largely tropical with two rainy seasons per year. In Lyantonde, the average rainfall ranges from 1000 to 1500 mm while the average temperature ranges from 18 °C in rainy season to 31.3 °C in dry season. Relatedly, Nakaseke has an average rainfall range of 1450 to 1500 mm and an average temperature range of 18 °C in rainy season to 30 °C in dry season.

**Fig. 1:**
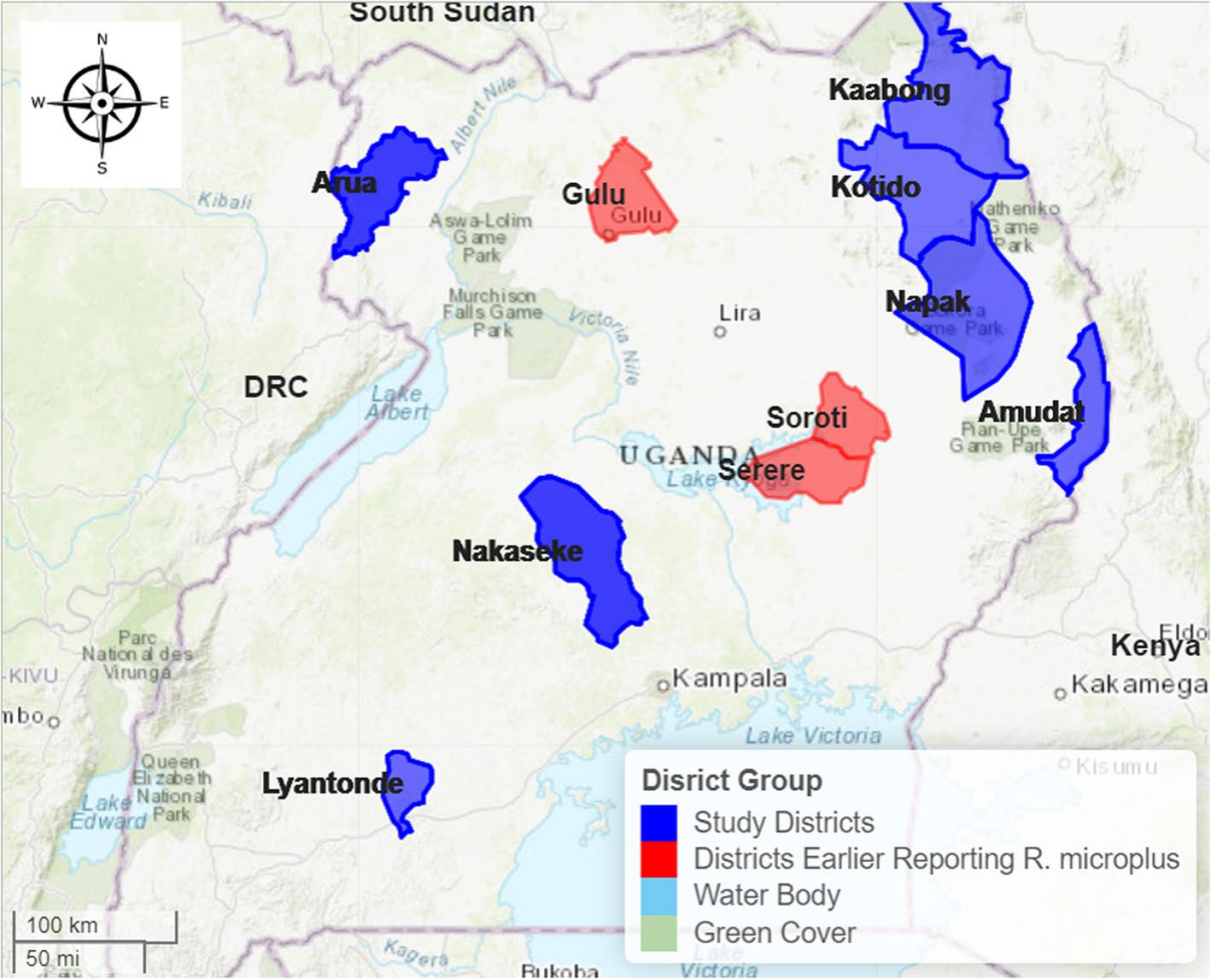
Map of Uganda showing study districts (The figure was generated by the authors in ArcMap 10.7 software using open-source shape files)

A stratified multi-stage selection strategy was used to identify the targeted locations for this study. Sampling frames (list of villages, parishes, and sub counties and districts) were obtained from respective district planning units. Using simple random sampling, target districts were selected. In each district, four sub-counties were selected and for each sub-county, two parishes were selected. One village was selected per parish, and a single kraal/herd sampled per village. In each sampling site, cattle were gathered in central cattle holding grounds or crushes. The cattle were then restrained while standing with aid of ropes and systematic sampling was used to select cows from which half body tick collections were done. Ideally, ticks were collected from about 20-24 cattle at each sampling site in seven districts (see Fig. 1).

### Tick collection and identification

Cattle were first restrained in a crush – where available or physically and half-body tick collections were undertaken. Only adult ticks (adults and nymphs) visible to the naked eye were removed using forceps and the collection was performed in about 25 minutes. Ticks were collected during the colder early morning hours of the day before departure of the animals for grazing (between 6 and 10 hrs). Ticks from each animal were placed in separate labeled glass vials and preserved with 70% ethanol. The vials were then transported in a cooler box to the Central Diagnostic Laboratory (CDL), Makerere University, Kampala, Uganda within seven days of collection. At CDL, ticks were identified to species level under a light stereomicroscope (Olympus™ SZ2-ST Stereomicroscope, Olympus Corporation, Tokyo, Japan), using morphological characteristics as previously described [37]. A morphologically and genetically validated *R. microplus* was photographed under a stereomicroscope (Olympus™ SZ2-ST Stereomicroscope, Olympus Corporation, Tokyo, Japan), (see Additional file 1). Ten *R. microplus* ticks from the Arua district and those of Karamoja region collections were selected for genetic validation of the morphological identification based on *12S* ribosomal RNA (12S rRNA), *16S* ribosomal RNA (16S rRNA) and the internal transcribed spacer 2 (ITS2) gene sequences [38].

### DNA extraction

Each tick was cleaned through five one-minute steps of centrifugation at 10,000 rpm in freshly prepared 1.5 ml of phosphate-buffered saline (PBS). Individual cleaned ticks wrapped in gold foil paper were made brittle by immersion in liquid nitrogen for 5 minutes and thereafter crushed with a sterile mortar and pestle to generate a tick homogenate. DNA was then extracted from each tick using the DNeasy® Blood and Tissue Kit (Qiagen, Hilden, Germany) according to the manufacturer’s instructions.

### DNA amplification

Molecular confirmation of *R. microplus* was based on the 12S ribosomal RNA (*12S rRNA*), 16S ribosomal RNA (*16S rRNA*) genes and the internal transcribed spacer 2 (*ITS2*) [38]. Conventional PCRs were performed on DNA extracted from morphologically identified *R. microplus* ticks with primers that targeted the two genes and ITS2 mentioned above. In all the PCRs, the reaction volume was 12.5 μl consisting of 6.25 μl PCR master mix, 0.25 μl primers and 5 μl of DNA template. The the target genes and their respective primers, expected amplicon sizes and the amplification conditions are shown in (Table 1). For each PCR, DreamTaq PCR Master Mix (2X, Thermo Scientific, Vilnius, Lithuania) was used. Five microliters of each PCR amplicon were run on 2% agarose gels to check the quality and yield of the PCR product, alongside a 1 kb DNA molecular ladder (Bioline, London, UK). PCR products were purified using the QIAquick PCR Purification Kit (Qiagen, Hilden, Germany) and Sanger sequenced in both forward and reverse directions at Inqaba Biotec (Pretoria, South Africa) using the ABI 3500XL Genetic Analyzer platform .

**Table 1:** The respective genes and their specific primers, expected PCR product sizes and the amplification conditions

Target gene	Primer	PCR product size (bp)	Reaction conditions	Reference
*12S rDNA*	F: 5’-GACACA GGAGGTAGTGA CAAG-3’ R: 5’-CTAAGA ATTTCA CCTCTG ACAGT-3’	320	94 °C 5 min, 35× (94 °C 30 s, 52 °C 45 s, 72 °C 45 s), 72 °C 7 min	[39]
*16S rDNA*	F: 5’- TTA AAT TGC TGT RGT ATT -3’ R: 5’- CCG GTC TGA ACT CASAWC -3’	455	94 °C 5 min, 35× (94 °C 30 s, 48 °C 45 s, 72 °C 45 s), 72 °C 7 min	[38]
*ITS2*	F: 5’-GAGTCTGCCAAATCCTTA C- 3’ R: 5’-TCCTCTACAGCTGCTTCG-3’	1200	94 °C 5 min, 35× (94 °C 30 s, 55 °C 45 s, 72 °C 90 s), 72 °C 7 min	[38]

### Gene sequence analysis

The *12S* rRNA, *16S* rRNA and ITS2 tick sequences from this study were queried in a Basic Local Alignment Tool (BLAST) search tool (https://blast.ncbi.nlm.nih.gov/Blast.cgi) to reveal their identity relative to published sequences. The identity of each sequence was assigned to the best hit of the tick species sequences returned with highest identity score (over 90%) and most significant E-value (closest to 0.0). The identified query sequences were submitted to the GenBank database. Thereafter, annotated sequences obtained from this study, and those downloaded from the GenBank database, were compiled and aligned using MUSCLE [40]. Phylogenetic analyses were performed using maximum likelihood method with 1000 bootstrap replication after best model of DNA evolution selected in MEGA 10 software [41]. To evaluate the evolutionary divergence of the queried sequences and those from GenBank, pairwise p-distance comparisons and calculations were completed using MEGA 10 software [41] using default settings for each sequence.

## Results

### Tick collections and identification with reference to the distribution of *R. microplus*

**Fig. 2:**
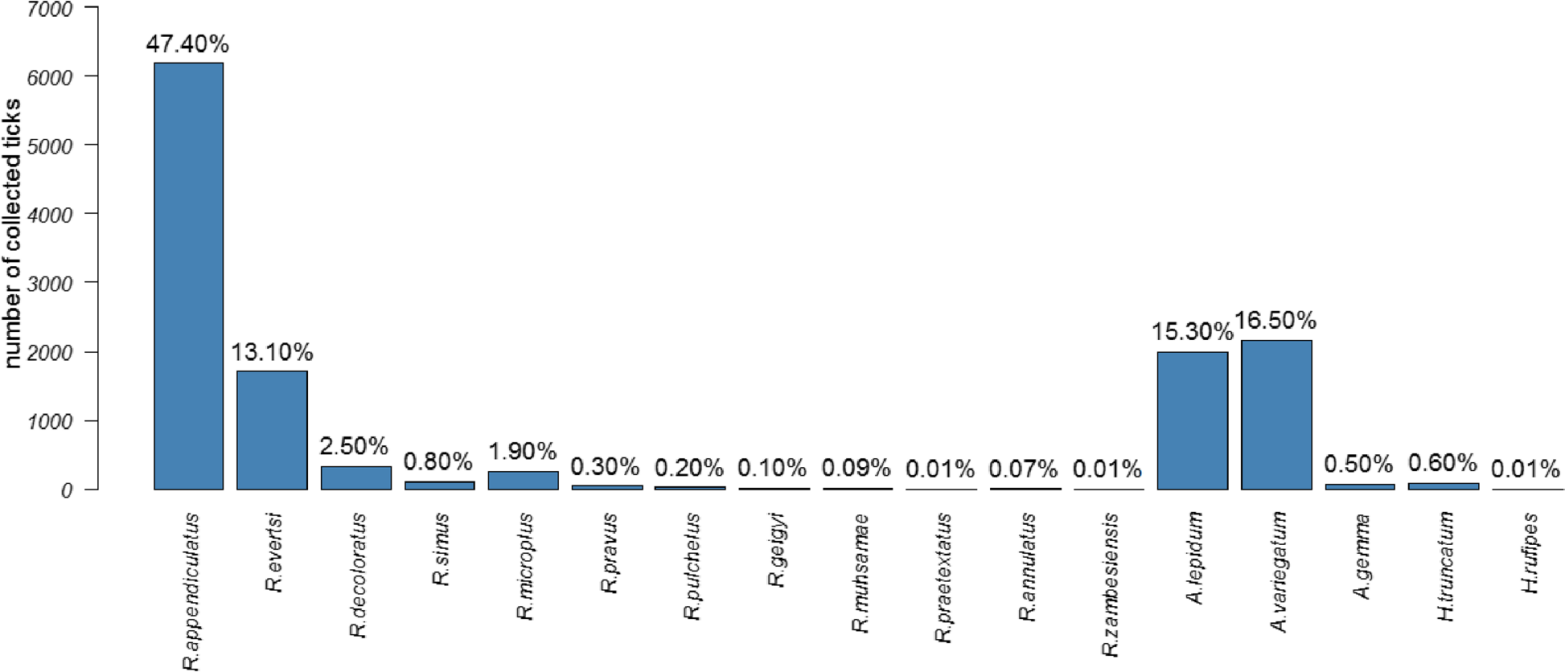
Tick species collected from cattle in the study districts. Percentages refer to the proportion for each species

Mature (adult and nymph) ticks (*n* = 13,019) were collected from 1,461 cattle and seventeen tick species were identified. The majority (47.4%; *n*=6184) of these ticks were *R. appendiculatus*. Other dominant tick species were *Amblyomma variegatum* (16.5%; *n*=2160), *A. lepidum* (15.3%; *n*=1997), *R. evertsi evertsi* (13.1%; *n*=1710), *R. decoloratus* (2.5%; *n*=334) and *R. microplus* (1.9%; *n*=257)*.* Of the 17 tick species identified in this tick collection, Karamoja region districts recorded 15, Arua district recorded ten while Lyantonde and Nakaseke districts recorded only two (see Fig. 2 and Additional file 2 for details).

A total of 257 *R. microplus* ticks were found infesting cattle in 18 of the 64 study sites. *Rhipicephalus microplus* ticks were found infesting cattle in the districts of Amudat, Kaabong, Napak (Karamoja region) and Arua (West Nile region). No *R. microplus* tick was recorded in the districts of Lyantonde, Nakaseke (Central region) and Kotido. In the district of Arua, *R. microplus* constituted 82.1% (*n*=211) of the collected ticks of the *Boophilus* sub-genus, this was distantly followed by the districts of Napak 16.3% (*n*=42), while Amudat and Kaabong recorded a sum of 1.5% (*n*=4). In Napak district, 85.7% (*n*=36) of the recorded *R. microplus* ticks were found infesting cattle in two adjacent villages of Iriiri Parish, Iriiri sub-county. Relatedly, in Arua district, *R. microplus* ticks were found infesting cattle in 12 of the 13 study sites. There was no major variation in the *R. microplus* tick counts in the 12 sites of Oniba, Yedu, Onguwa, Alivu, Odranyiri, Eraka, Kova, Ego-Ayiko B, Oleba, Amakuva, Elikoa and Ego-Ayiko A (see Table 2).

**Table 2:** Study sites where *R. microplus* was found, the corresponding number of other *Rhipicephalus* (*Boophilus*) tick species identified and the percentage proportion of *R.microplus* and *R.decoloratus* ticks in relation to total tick collection per study site

District	Sub county	Village	Ticks collected	Rm	Rd	Rg	Ra	% of total tick collection in that study site	
								Rm	Rd
Napak	Matany	Arecheck	501	1	40	4	0	0.1	7.9
	Lopeii	Nakatiyat	142	5	1	0	0	3.5	0.7
	Iriiri	Ariama-aokot	442	20	18	1	0	4.5	4.0
	Iriiri	Nakwakwa	245	16	30	0	0	6.5	12.2
Kaabong	Kathile south	Kamacharikol	322	1	18	4	0	0.3	5.5
	Sidok	Karichor	318	1	16	0	0	0.3	5.0
Amudat	Looro	Murut	553	2	3	0	0	0.3	0.5
Arua	Adumi	Oniba	302	16	0	0	0	5.2	0
	Adumi	Yedu	231	28	0	0	0	12.1	0
	Adumi	Onguwa	792	28	0	0	0	3.5	0
	Adumi	Alivu	297	12	7	0	0	4.0	2.3
	Adumi	Odranyiri	381	13	9	0	1	3.4	2.3
	Adumi	Eraka	210	7	5	0	2	3.3	2.3
	Adumi	Kova	425	16	0	0	0	3.7	0
	Adumi	Ego-Ayiko B	443	25	0	2	0	5.6	0
	Adumi	Oleba	228	36	1	0	7	15.7	0.4
	Adumi	Oliva-Aderi	80	0	0	0	0	0	0
	Adumi	Amakuva	110	6	4	1	0	5.4	3.6
	Adumi	Elikoa	197	8	1	0	0	4.0	0.5
	Adumi	Ego-Ayiko A	249	16	0	0	0	6.4	0

*Rm* Rhipicephalus microplus, *Rd* Rhipicephalus decoloratus, *Rg* Rhipicephalus geigyi, *Ra* Rhipicephalus annulatus

**Fig. 3:**
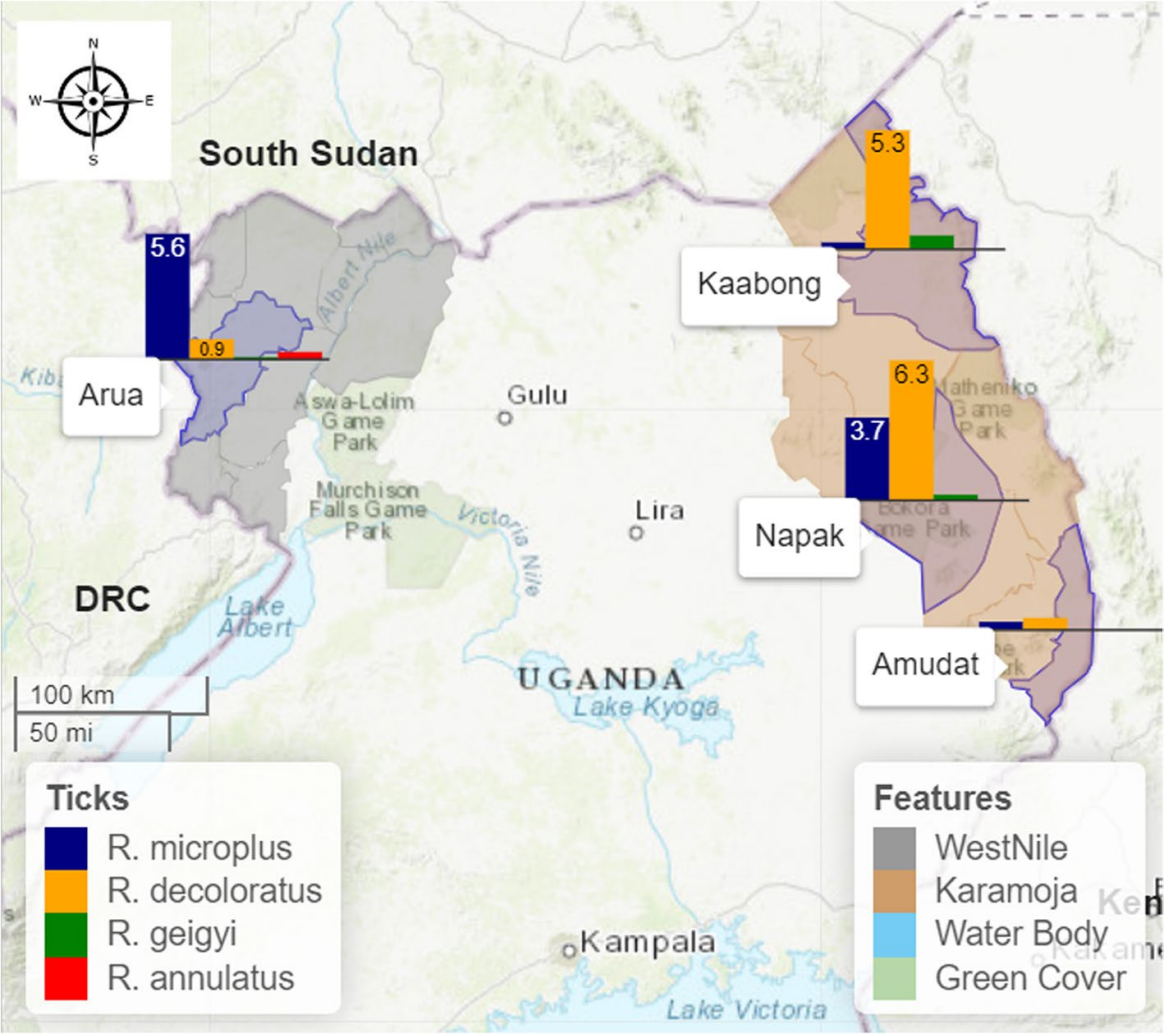
Map showing study sites that recorded the *Rhipicephalus* (*Boophilus*) tick species and bar charts with their percentage dominance per study site (The figure was generated by the authors in ArcMap 10.7 software using open-source shape files)

Four *Rhipicephalus* (*Boophilus*) tick species were recorded in this study; that is *R. microplus*, *R. decoloratus*, *R. geigyi* and *R. annulatus*. Arua district had all the four species infesting cattle, while the districts of Karamoja region did not record a single *R. annulatus.* In Central region (Lyantonde and Nakaseke districts), only *R. decoloratus* was recorded. Of the total 618 *Rhipicephalus* (*Boophilus*) tick species recorded, *R. decoloratus* accounted for 50.04% (*n*=334), followed by *R. microplus* at 41.58% (*n*=257), *R. geigyi* at 2.75% (*n*=17) and *R. annulatus* at 1.61% (*n*=10). In the districts of Amudat, Kaabong and Napak, *R. decoloratus* was the most dominant (76.1%; *n*=179) of the three *Rhipicephalus* (*Boophilus*) species recorded followed by *R. microplus* (19.5%; *n*=46) and *R. geigyi* (4.2%; *n*=10). Contrariwise, *R. microplus* was more dominant (84%; *n*=211) in Arua District followed by *R. decoloratus* (10.7%; *n*=27), *R. annulatus* (3.9%; *n*=10) and *R. geigyi* (1.1%; *n*=3), (see Fig. 3).

*Rhipicephalus microplus* and *R. decoloratus* co-existed in 46% of the 13 study sites in Arua district, but *R. microplus* counts accounted for 88.6% of the total (*n*=238) identified *R. microplus* and *R. decoloratus* ticks. Relatedly, 46% of the 13 study sites only recorded *R. microplus* ticks and not a single *R. decoloratus* tick. In the districts of Amudat, Kaabong and Napak, *R. microplus* and *R. decoloratus* ticks co-existed in 23.3% of the 30 study sites; however, *R. decoloratus* counts accounted for 79.5% of the total (*n*=225) identified *R. microplus* and *R. decoloratus* ticks.

### Molecular identification of *R. microplus*

**Fig. 4:**
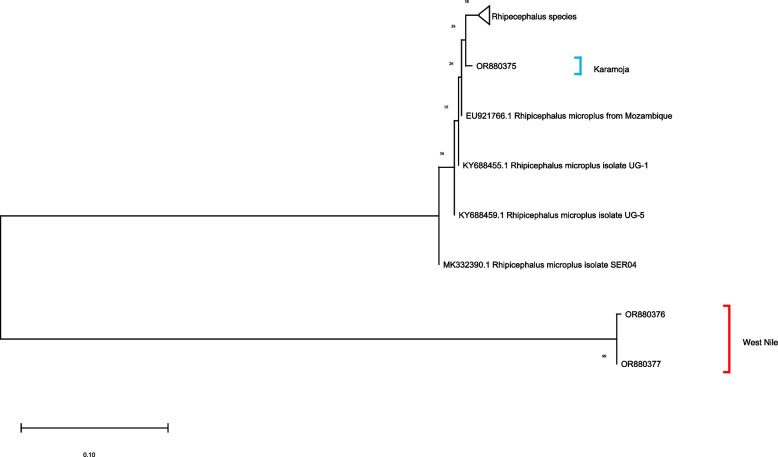
Maximum likelihood phylogenetic analysis of 12S rRNA gene sequences of *R.microplus* ticks. Sequences generated from this study are marked as Karamoja and West Nile

**Fig. 5:**
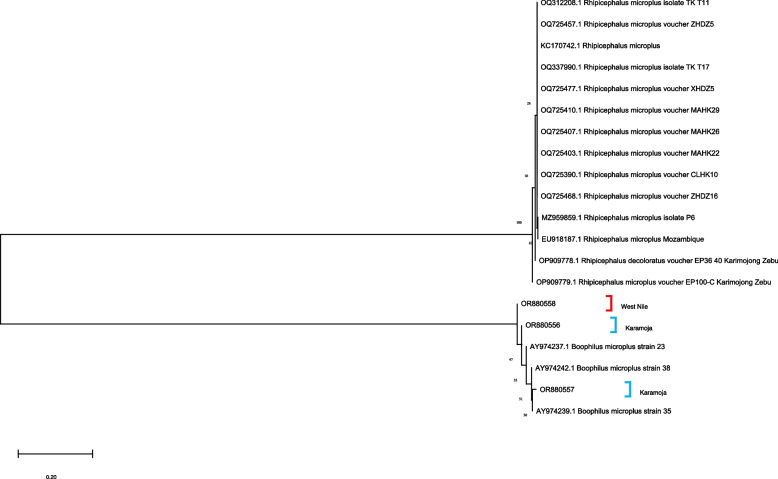
Maximum likelihood phylogenetic analysis of 16S rRNA gene sequences of *R.microplus* ticks. Sequences generated from this study are marked as Karamoja and West Nile

**Fig. 6:**
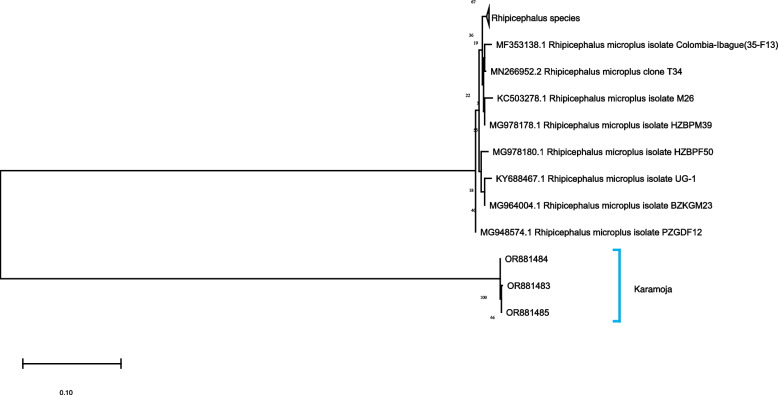
Maximum likelihood phylogenetic analysis of ITS2 spacer of the rRNA gene sequences of *R.microplus* of ticks. Sequences generated from this study are marked as Karamoja

Ten of the 257 ticks identified using morpho-taxonomic keys as *R. microplus* were further analysed and confirmed by assessing the sequences of their *12S* rRNA, *16S* rRNA and *ITS2* regions. The identified query sequences from this study were submitted to the GenBank database and allocated following accession numbers: OR880375, OR880376, OR880377, OR880556, OR880557, OR880558, OR881483, OR881484 and OR881485 (see Additional file 3). Phylogenetic analyses of the *12S* rRNA (Fig. 4), *16S* rRNA (Fig. 5) and *ITS2* (Fig. 6) regions revealed subgrouping with *R. microplus* collected from other parts of the world.

## Discussion

The tick *R. microplus*, also known as the Asian blue tick is the most widely distributed ixodid infesting cattle globally [2, 12, 13]. This tick is invasive and has a tendency to displace other ticks of the same sub-genus such as *R. decoloratus* and *R. geigyi* [2]. In Uganda, this tick was first reported in 2020 in Serere district, and also in Soroti and Gulu districts [6, 23] while, in the current report, this tick was found infesting cattle in Amudat, Kaabong, Napak and Arua districts. Unlike the previous reports where this tick had displaced *R. decoloratus* completely in Serere district [23], in the current report the tick has been found to be co-existing with the indigenous *R. decoloratus* except for Arua district where its seems to be displacing *R. decoloratus*.

*Rhipicephalus microplus* ticks were found infesting cattle in 18 study sites in four of the seven study districts compared to only six sites in Serere district, five sites in Soroti and Gulu districts respectively. In the previous reports 687, 23 and 13 *R. microplus* ticks were found infesting cattle in Serere, Soroti and Gulu districts respectively [6, 23]. In comparison, the current study recorded only 257 *R. microplus* ticks infesting cattle in four districts and the majority (82%, *n*=211) were found infesting cattle in Amudat district. The increasing number of districts reporting *R. microplus* is evidence of its invasive nature. This tick may colonise and establish itself well in Uganda given its invasive nature and the combination of poor control of animal movement and communal grazing practices. The number of *R. microplus* and the displacement of the indigenous *R. decoloratus* can give an indication of the duration of the tick infestation in a new area [25].

Much as the majority of the *Rhipicephalus* (*Boophilus*) ticks collected and identified in this study are *R. decoloratus* (52.8%; *n*=334), the counts of *R. microplus* are equally high (41.5%; *n*=257), followed *R. geigyi* (2.6%; *n*=13) and *R. annulatus* (2%; *n*=10). The proportion of the *R. microplus* ticks among the *Rhipicephalus* (*Boophilus*) tick species collected in areas where *R. microplus* has been reported is changing significantly. In Serere district, it was the only *Rhipicephalus* (*Boophilus*) tick species found at a 100% dominance having possibly out survived the indigenous *R. decoloratus* [23]. In Soroti and Gulu districts, *R. microplus* coexisted with *R. decoloratus* at a proportion of 41% and 41.9% for Soroti and Gulu districts respectively [6]. While as reported in this study, the proportion of *R. microplus* among the tick counts of the three *Rhipicephalus* (*Boophilus*) tick species collected in the districts of Amudat, Kaabong and Napak districts was 16.6%, 3.5% and 25.1% respectively. Arua district recorded four *Rhipicephalus* (*Boophilus*) tick species and the proportion of *R. microplus* was 84%. The high variance (3.5% - 84%) of the proportion of *R. microplus* among the *Rhipicephalus* (*Boophilus*) tick species collected in this study is evidence of its invasiveness and displacement behaviour [2, 11, 26, 27].

*Rhipicephalus microplus* was most probably introduced into Uganda through animal importation [2] and cross-border cattle movement for trade and through cattle rustling, especially in the semi-arid north-eastern region of Uganda [42]. The co-occurrence of *R. decoloratus* and *R. microplus* in 12 of the 18 study sites suggests that these are sites of recent introduction of this tick [25]. However, in Arua District, *R. decoloratus* had been displaced completely in six of the study sites that recorded *R. microplus*, a feat that takes years to achieve [25]. The mechanism of displacement of one *Boophilus* tick by another as has been reported in Uganda [23], South Africa [27], Zambia [43] and West African countries [2, 11, 26] is still unclear. It is postulated to be either due sterile off-springs of interspecific mating [44], a faster life-cycle of *R. microplus* compared to *R. decoloratus,* because of its high success rate of feeding on cattle [45] or because of the high degree of development of resistance to acaricides by *R. microplus* [28, 32].

A diversity of tick species has been found to infest cattle in Uganda [3–7]. Much as there is a significant variation in the tick population structure between geographical regions of the country - due to changes in microclimate and vegetation - *R. appendiculatus* remains the predominant tick species in this survey as previously reported [3, 5, 6, 23]. Other dominant tick species infesting cattle, like *A. variegatum*, *A. lepidum*, *R. decoloratus*, and *R. evertsi* vector some of the major diseases such as anaplasmosis, babesiosis and heartwater. This validates the belief by most livestock keeping communities that ticks and associated diseases are a key constraint to livestock production [46, 47] .

Molecular phylogenetic analyses of the *12S* rRNA, *16S* rRNA genes and ITS2 region of the tick isolate from Arua District and Karamoja region revealed different sequence variants. This could suggest that the *R. microplus* ticks in the two regions were introduced from different sources [48]. This validates the assertion that un-controlled cattle movement and the importation of livestock either through trade or cross-border livestock movement, separately influence the spread of *R. microplus* [2, 10, 11].

Much as the habitat and distribution of *R. geigyi* is reported to include Uganda and Sudan [37], this tick has not been reported in previous tick surveillance studies carried out in Uganda [3–8, 49]. It has a limited distribution mainly confined to West Africa. Cross border cattle movement especially in the Karamoja region and the influx of refugees with their livestock to West Nile region of Uganda could have introduced this tick from Sudan where it was earlier found infesting livestock and some wild life [50]. Likewise, *R. annulatus* has not been reported in past tick survey studies carried out in Uganda [3–8, 49]. Its habitat and distribution did not include Uganda [37]. It is mainly a tick of West and North Africa but also found in South Sudan, Central African Republic and Democratic republic of Congo. The influx of refugees from South Sudan to West Nile region with their livestock could be a more probable route of introduction of this tick to Arua district. The tick had earlier been reported to infest livestock in the Anglo-Egyptian area – the present day Egypt and Sudan [51].

The spread of *R. microplus* will negatively affect the livestock industry in Uganda. Apart from being an efficient vector of the more pathogenic *B. bovis* [52], *R. microplus* tends to quickly develop resistance to acaricides [28]. Given the current poorly regulated acaricide usage, which is characterized by misuse and overuse of anti-insect and anti-tick chemicals [30], there is a high likelihood that *R. microplus* will develop resistance to acaricides [32].

Resistance to acaricides is postulated to be one of the mechanisms through which this tick is able to rapidly invade new areas and displace other tick species of the *Boophilus* subgenus [2]. Therefore, there is a likelihood of the rapid spread of this tick throughout the country, and the associated occurrence of severe losses in the livestock sector. Country-wide surveillance and molecular studies should be carried to determine the extent of *R. microplus* spread and to understand the factors that are responsible for its persistence in areas. Regulation of livestock movement and usage of acaricides must be stepped up so as to minimize the spread of this tick and its pathogen *B. bovis*. Livestock farmers should be sensitized on the likely impact of *R. microplus* and *B. bovis* to their livestock and on what they should do to minimize the spread of this tick across the country.

## Conclusion

This study found *R. microplus* ticks infesting cattle in four districts during a survey conducted in three regions of Uganda. The complete absence of *R. decoloratus*, an indigenous tick, in six sites in Arua District probably suggests its displacement by *R. microplus*. *Rhipicephalus microplus* negatively affects livestock production and transmits pathogens of veterinary importance. This study also found *R.annulatus* and *R.geigyi* tick species that were previously not reported in Uganda. There is a need for further surveillance activities and molecular analysis of *R. microplus* ticks to determine their distribution and to deepen our understanding of the ecological scenarios that lead to tick persistence.

### Supplementary Information

**Supplementary Material 1. **

**Supplementary Material 2. **

**Supplementary Material 3. **

## Data Availability

Data supporting the conclusion of this article are included within the article. The newly generated tick sequences were submitted to the GenBank database under the accession numbers (OR880375, OR880376, OR880377, OR880556, OR880557, OR880558, OR881483, OR881484 and OR881485). The datasets used and/or analyses during the preset study are available from the corresponding author upon reasonable request.

## Abbreviations

*12S* rRNA: *12**S* ribosomal RNA gene
*16S* rRNA: *16**S* ribosomal RNA gene
ITS2: internal transcribed spacer 2
DNA: deoxyribonucleic acid
RNA: ribonucleic acid
GPS: geographical positioning system
TTBDs: ticks and tick-borne disease
NARO: National Agricultural Research Organization
