# Supplementary material for: Identification and distribution of Rhipicephalus microplus in selected high-cattle density districts in Uganda: signaling future demand for novel tick control approaches
Source: BMC Vet Res. 2024 Mar 25;20:119. doi: 10.1186/s12917-024-03979-z (PMC10964625; doi:10.1186/s12917-024-03979-z)
Supplement: Supplementary file 1 — Supplementary Material 1. [file 12917_2024_3979_MOESM1_ESM.pdf]

Additional file 1: Figure S1. *Rhipicephalus microplus* ventral views.

Rhipicephalus microplus

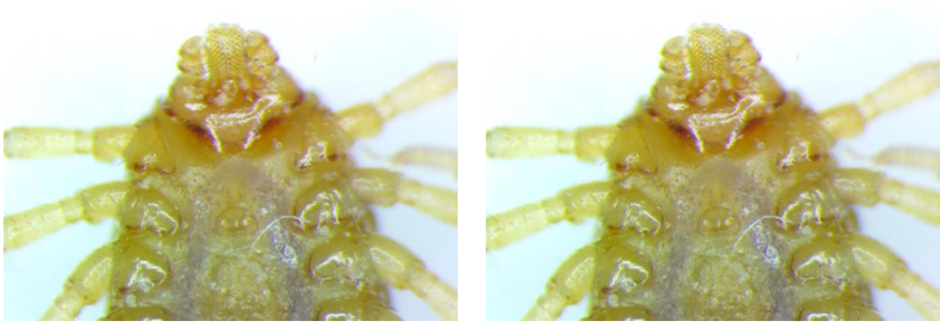

4 + 4 collumn arrangement of the Hypostomol teeth

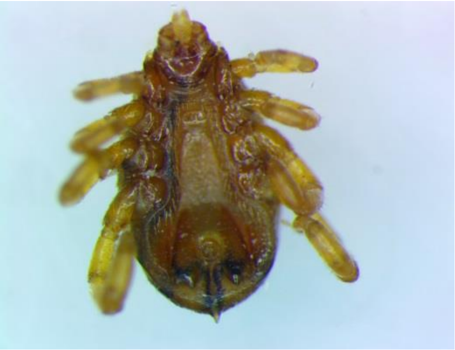

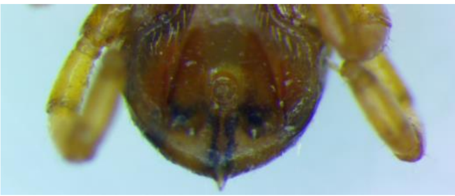

Small adenal plates and
Broad U shaped genital aperture

Caudal appendage
