# Supplementary material for: Identification and distribution of Rhipicephalus microplus in selected high-cattle density districts in Uganda: signaling future demand for novel tick control approaches
Source: BMC Vet Res. 2024 Mar 25;20:119. doi: 10.1186/s12917-024-03979-z (PMC10964625; doi:10.1186/s12917-024-03979-z)
Supplement: Supplementary file 2 — Supplementary Material 2. [file 12917_2024_3979_MOESM2_ESM.docx]

**Additional file 2: Table S1:** Distribution of the identified tick species per district

| Tick genus | Tick species | No. of ticks collected (% of total collection) | Amudat District | Kaabong District | Kotido District | Napak District | Lyantonde District | Nakaseke District | Arua District |
| --- | --- | --- | --- | --- | --- | --- | --- | --- | --- |
| *Rhipicephalus* | *R.appendiculatus* | 6184 | 709 | 179 | 550 | 984 | 547 | 1817 | 1398 |
|  |  | (47.4) |  |  |  |  |  |  |  |
|  | *R.evertsi* | 1710 | 71 | 449 | 273 | 370 | 0 | 0 | 547 |
|  |  | (13.1) |  |  |  |  |  |  |  |
|  | *R.decoloratus* | 334 | 10 | 50 | 8 | 119 | 78 | 42 | 27 |
|  |  | (2.5) |  |  |  |  |  |  |  |
|  | *R.simus* | 106 | 6 | 53 | 34 | 13 | 0 | 0 | 0 |
|  |  | (0.8) |  |  |  |  |  |  |  |
|  | *R.microplus* | 257 | 2 | 2 | 0 | 42 | 0 | 0 | 211 |
|  |  | (1.9) |  |  |  |  |  |  |  |
|  | *R.pravus* | 41 | 1 | 8 | 22 | 2 | 0 | 0 | 8 |
|  |  | (0.3) |  |  |  |  |  |  |  |
|  | *R.pulchelus* | 27 | 25 | 0 | 2 | 0 | 0 | 0 | 0 |
|  |  | (0.2) |  |  |  |  |  |  |  |
|  | *R.geigyi* | 17 | 0 | 4 | 4 | 6 | 0 | 0 | 3 |
|  |  | (0.1) |  |  |  |  |  |  |  |
|  | *R.muhsamae* | 12 | 0 | 4 | 8 | 0 | 0 | 0 | 0 |
|  |  | (0.09) |  |  |  |  |  |  |  |
|  | *R.praetextatus* | 1 | 1 | 0 | 0 | 0 | 0 | 0 | 0 |
|  |  | (0.007) |  |  |  |  |  |  |  |
|  | *R.annulatus* | 10 | 0 | 0 | 0 | 0 | 0 | 0 | 10 |
|  |  | (0.07) |  |  |  |  |  |  |  |
|  | *R.zambesiensis* | 2 | 0 | 0 | 0 | 0 | 0 | 0 | 2 |
|  |  | (0.01) |  |  |  |  |  |  |  |
| *Amblyomma* | *A.lepidum* | 1997 | 428 | 27 | 759 | 783 | 0 | 0 | 0 |
|  |  | (15.3) |  |  |  |  |  |  |  |
|  | *A.variegatum* | 2160 | 148 | 774 | 107 | 235 | 0 | 0 | 896 |
|  |  | (16.5) |  |  |  |  |  |  |  |
|  | *A.gemma* | 68 | 68 | 0 | 0 | 0 | 0 | 0 | 0 |
|  |  | (0.5) |  |  |  |  |  |  |  |
| *Hyaloma* | *H.truncatum* | 91 | 18 | 48 | 15 | 10 | 0 | 0 | 0 |
|  |  | (0.6) |  |  |  |  |  |  |  |
|  | *H.rufipes* | 2 | 0 | 0 | 1 | 0 | 0 | 0 | 1 |
|  |  | (0.01) |  |  |  |  |  |  |  |
| Totals | | 13019 | 1487 | 1598 | 1783 | 2564 | 625 | 1859 | 3103 |
